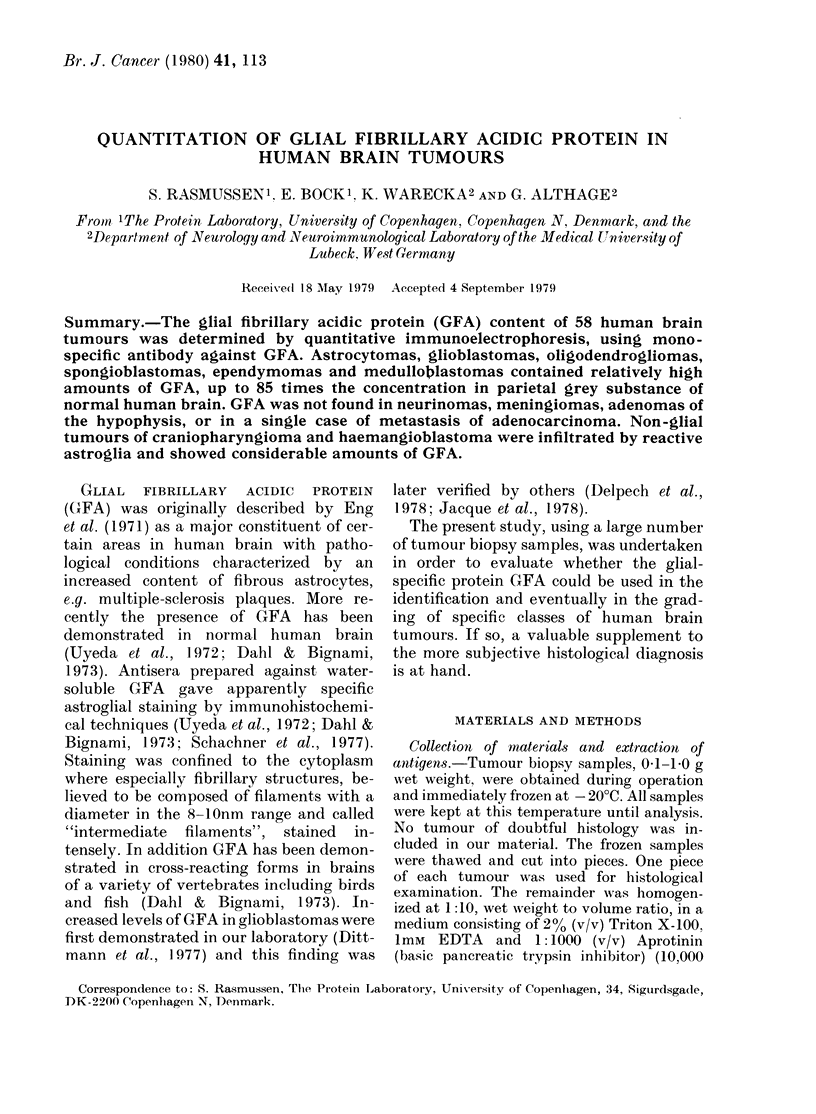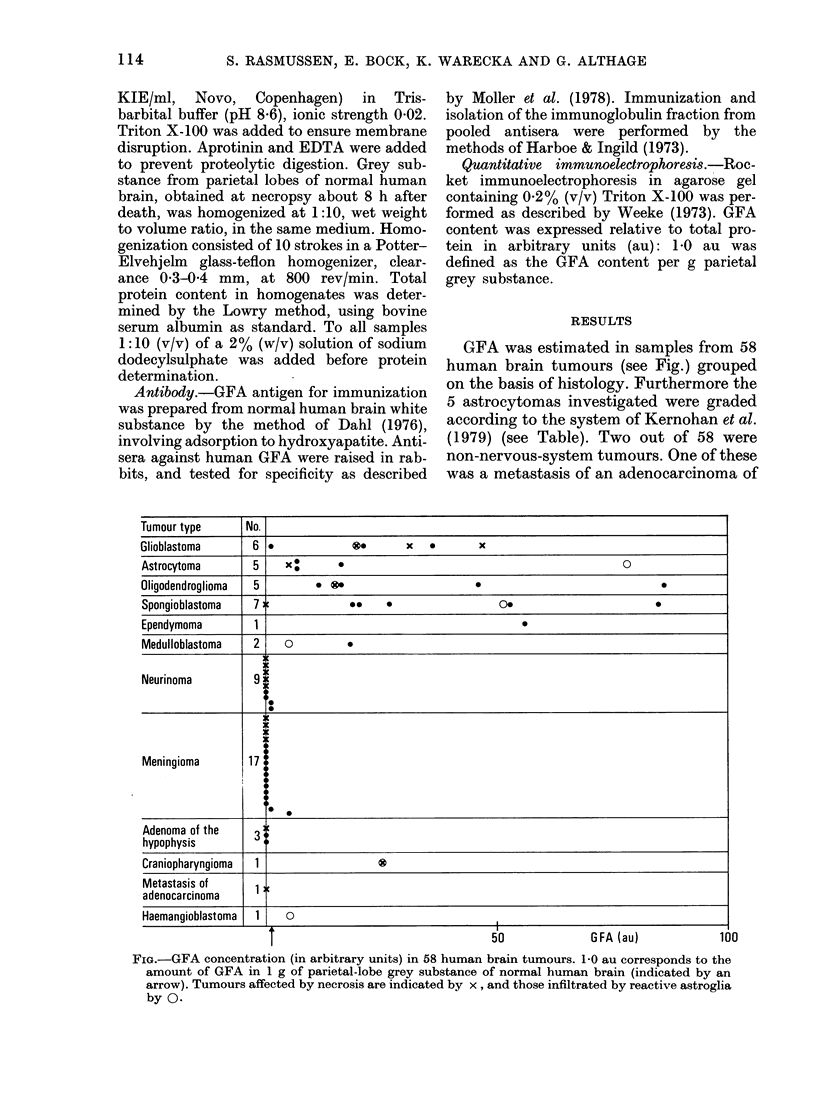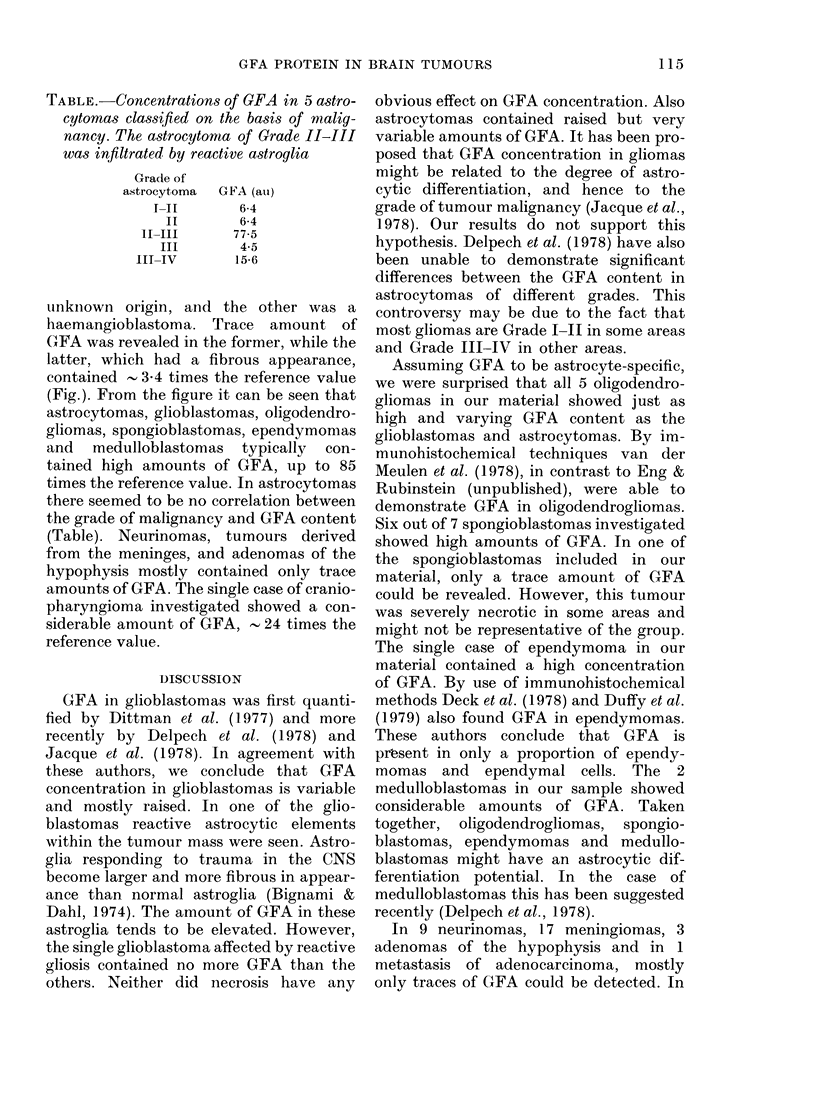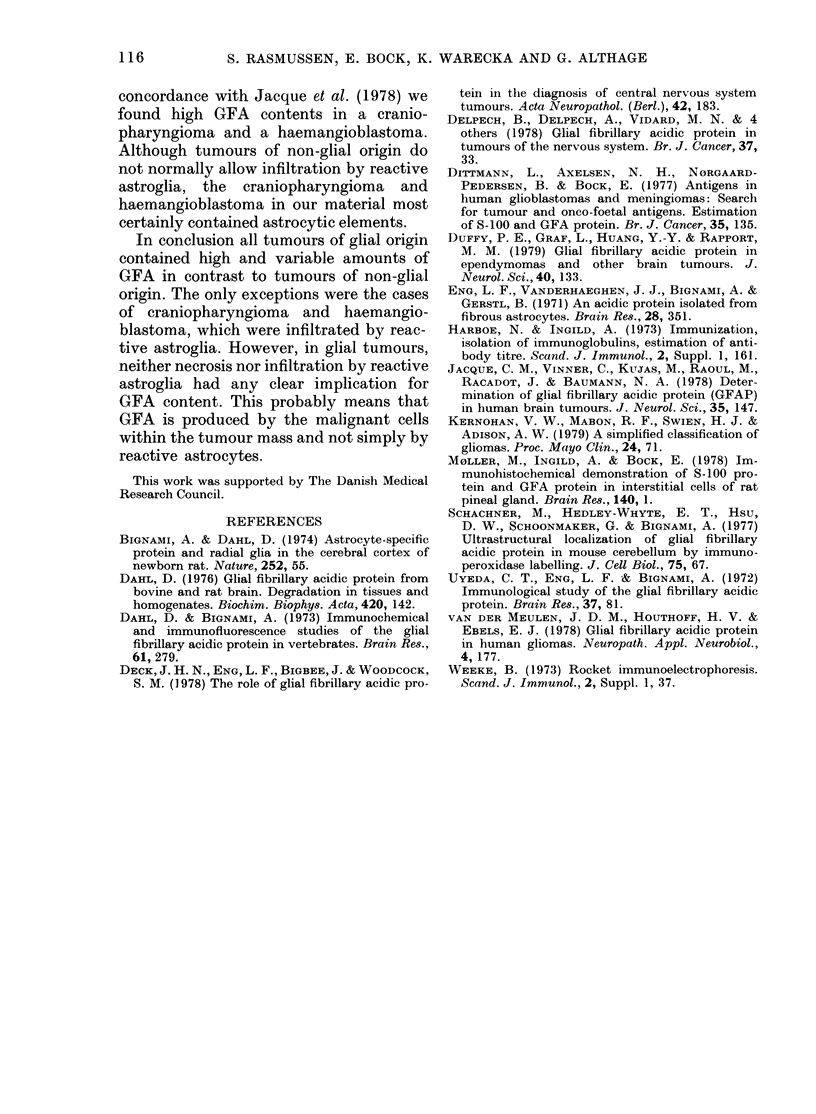# Quantitation of glial fibrillary acidic protein in human brain tumours.

**DOI:** 10.1038/bjc.1980.12

**Published:** 1980-01

**Authors:** S. Rasmussen, E. Bock, K. Warecka, G. Althage

## Abstract

The glial fibrillary acidic protein (GFA) content of 58 human brain tumours was determined by quantitative immunoelectrophoresis, using monospecific antibody against GFA. Astrocytomas, glioblastomas, oligodendrogliomas, spongioblastomas, ependymomas and medulloblastomas contained relatively high amounts of GFA, up to 85 times the concentration in parietal grey substance of normal human brain. GFA was not found in neurinomas, meningiomas, adenomas of the hypophysis, or in a single case of metastasis of adenocarcinoma. Non-glial tumours of craniopharyngioma and haemangioblastoma were infiltrated by reactive astroglia and showed considerable amounts of GFA.


Br. J. Cancer (1980) 41, 113

### QUANTITATION OF GLIAL FIBRILLARY ACIDIC PROTEIN IN HUMAN BRAIN TUMOURS

S. RASMUSSEN[1], E. BOCK[1], K. WARECKA[2] AND G. ALTHAGE[2]

*From [1]The Protein Laboratory, University of Copenhagen, Copenhagen N, Denmark, and the [2]Department of Neurology and Neuroimmunological Laboratory of the Medical University of Lubeck, West Germany*

Received 18 May 1979 Accepted 4 September 1979

**Summary.—The glial fibrillary acidic protein (GFA) content of 58 human brain tumours was determined by quantitative immunoelectrophoresis, using monospecific antibody against GFA. Astrocytomas, glioblastomas, oligodendrogliomas, spongioblastomas, ependymomas and medulloblastomas contained relatively high amounts of GFA, up to 85 times the concentration in parietal grey substance of normal human brain. GFA was not found in neurinomas, meningiomas, adenomas of the hypophysis, or in a single case of metastasis of adenocarcinoma. Non-glial tumours of craniopharyngioma and haemangioblastoma were infiltrated by reactive astroglia and showed considerable amounts of GFA.**

GLIAL FIBRILLARY ACIDIC PROTEIN (GFA) was originally described by Eng *et al.* (1971) as a major constituent of certain areas in human brain with pathological conditions characterized by an increased content of fibrous astrocytes, *e.g.* multiple-sclerosis plaques. More recently the presence of GFA has been demonstrated in normal human brain (Uyeda *et al.*, 1972; Dahl & Bignami, 1973). Antisera prepared against water-soluble GFA gave apparently specific astroglial staining by immunohistochemical techniques (Uyeda *et al.*, 1972; Dahl & Bignami, 1973; Schachner *et al.*, 1977). Staining was confined to the cytoplasm where especially fibrillary structures, believed to be composed of filaments with a diameter in the 8–10nm range and called "intermediate filaments", stained intensely. In addition GFA has been demonstrated in cross-reacting forms in brains of a variety of vertebrates including birds and fish (Dahl & Bignami, 1973). Increased levels of GFA in glioblastomas were first demonstrated in our laboratory (Dittmann *et al.*, 1977) and this finding was later verified by others (Delpech *et al.*, 1978; Jacque *et al.*, 1978).

The present study, using a large number of tumour biopsy samples, was undertaken in order to evaluate whether the glial-specific protein GFA could be used in the identification and eventually in the grading of specific classes of human brain tumours. If so, a valuable supplement to the more subjective histological diagnosis is at hand.

### MATERIALS AND METHODS

*Collection of materials and extraction of antigens.*—Tumour biopsy samples, 0·1–1·0 g wet weight, were obtained during operation and immediately frozen at −20°C. All samples were kept at this temperature until analysis. No tumour of doubtful histology was included in our material. The frozen samples were thawed and cut into pieces. One piece of each tumour was used for histological examination. The remainder was homogenized at 1:10, wet weight to volume ratio, in a medium consisting of 2% (v/v) Triton X-100, 1mM EDTA and 1:1000 (v/v) Aprotinin (basic pancreatic trypsin inhibitor) (10,000

Correspondence to: S. Rasmussen, The Protein Laboratory, University of Copenhagen, 34, Sigurdsgade, DK-2200 Copenhagen N, Denmark.

KIE/ml, Novo, Copenhagen) in Tris-barbital buffer (pH 8·6), ionic strength 0·02. Triton X-100 was added to ensure membrane disruption. Aprotinin and EDTA were added to prevent proteolytic digestion. Grey substance from parietal lobes of normal human brain, obtained at necropsy about 8 h after death, was homogenized at 1:10, wet weight to volume ratio, in the same medium. Homogenization consisted of 10 strokes in a Potter–Elvehjelm glass-teflon homogenizer, clearance 0·3–0·4 mm, at 800 rev/min. Total protein content in homogenates was determined by the Lowry method, using bovine serum albumin as standard. To all samples 1:10 (v/v) of a 2% (w/v) solution of sodium dodecylsulphate was added before protein determination.

*Antibody.*—GFA antigen for immunization was prepared from normal human brain white substance by the method of Dahl (1976), involving adsorption to hydroxyapatite. Antisera against human GFA were raised in rabbits, and tested for specificity as described by Moller *et al.* (1978). Immunization and isolation of the immunoglobulin fraction from pooled antisera were performed by the methods of Harboe & Ingild (1973).

*Quantitative immunoelectrophoresis.*—Rocket immunoelectrophoresis in agarose gel containing 0·2% (v/v) Triton X-100 was performed as described by Weeke (1973). GFA content was expressed relative to total protein in arbitrary units (au): 1·0 au was defined as the GFA content per g parietal grey substance.

### RESULTS

GFA was estimated in samples from 58 human brain tumours (see Fig.) grouped on the basis of histology. Furthermore the 5 astrocytomas investigated were graded according to the system of Kernohan *et al.* (1979) (see Table). Two out of 58 were non-nervous-system tumours. One of these was a metastasis of an adenocarcinoma of

FIG.—GFA concentration (in arbitrary units) in 58 human brain tumours. 1·0 au corresponds to the amount of GFA in 1 g of parietal-lobe grey substance of normal human brain (indicated by an arrow). Tumours affected by necrosis are indicated by ×, and those infiltrated by reactive astroglia by ○.

TABLE.—*Concentrations of GFA in 5 astrocytomas classified on the basis of malignancy. The astrocytoma of Grade II–III was infiltrated by reactive astroglia*

| Grade of astrocytoma | GFA (au) |
|---|---|
| I–II | 6·4 |
| II | 6·4 |
| II–III | 77·5 |
| III | 4·5 |
| III–IV | 15·6 |

unknown origin, and the other was a haemangioblastoma. Trace amount of GFA was revealed in the former, while the latter, which had a fibrous appearance, contained ~3·4 times the reference value (Fig.). From the figure it can be seen that astrocytomas, glioblastomas, oligodendrogliomas, spongioblastomas, ependymomas and medulloblastomas typically contained high amounts of GFA, up to 85 times the reference value. In astrocytomas there seemed to be no correlation between the grade of malignancy and GFA content (Table). Neurinomas, tumours derived from the meninges, and adenomas of the hypophysis mostly contained only trace amounts of GFA. The single case of craniopharyngioma investigated showed a considerable amount of GFA, ~24 times the reference value.

### DISCUSSION

GFA in glioblastomas was first quantified by Dittman *et al.* (1977) and more recently by Delpech *et al.* (1978) and Jacque *et al.* (1978). In agreement with these authors, we conclude that GFA concentration in glioblastomas is variable and mostly raised. In one of the glioblastomas reactive astrocytic elements within the tumour mass were seen. Astroglia responding to trauma in the CNS become larger and more fibrous in appearance than normal astroglia (Bignami & Dahl, 1974). The amount of GFA in these astroglia tends to be elevated. However, the single glioblastoma affected by reactive gliosis contained no more GFA than the others. Neither did necrosis have any obvious effect on GFA concentration. Also astrocytomas contained raised but very variable amounts of GFA. It has been proposed that GFA concentration in gliomas might be related to the degree of astrocytic differentiation, and hence to the grade of tumour malignancy (Jacque *et al.*, 1978). Our results do not support this hypothesis. Delpech *et al.* (1978) have also been unable to demonstrate significant differences between the GFA content in astrocytomas of different grades. This controversy may be due to the fact that most gliomas are Grade I–II in some areas and Grade III–IV in other areas.

Assuming GFA to be astrocyte-specific, we were surprised that all 5 oligodendrogliomas in our material showed just as high and varying GFA content as the glioblastomas and astrocytomas. By immunohistochemical techniques van der Meulen *et al.* (1978), in contrast to Eng & Rubinstein (unpublished), were able to demonstrate GFA in oligodendrogliomas. Six out of 7 spongioblastomas investigated showed high amounts of GFA. In one of the spongioblastomas included in our material, only a trace amount of GFA could be revealed. However, this tumour was severely necrotic in some areas and might not be representative of the group. The single case of ependymoma in our material contained a high concentration of GFA. By use of immunohistochemical methods Deck *et al.* (1978) and Duffy *et al.* (1979) also found GFA in ependymomas. These authors conclude that GFA is present in only a proportion of ependymomas and ependymal cells. The 2 medulloblastomas in our sample showed considerable amounts of GFA. Taken together, oligodendrogliomas, spongioblastomas, ependymomas and medulloblastomas might have an astrocytic differentiation potential. In the case of medulloblastomas this has been suggested recently (Delpech *et al.*, 1978).

In 9 neurinomas, 17 meningiomas, 3 adenomas of the hypophysis and in 1 metastasis of adenocarcinoma, mostly only traces of GFA could be detected. In concordance with Jacque *et al.* (1978) we found high GFA contents in a craniopharyngioma and a haemangioblastoma. Although tumours of non-glial origin do not normally allow infiltration by reactive astroglia, the craniopharyngioma and haemangioblastoma in our material most certainly contained astrocytic elements.

In conclusion all tumours of glial origin contained high and variable amounts of GFA in contrast to tumours of non-glial origin. The only exceptions were the cases of craniopharyngioma and haemangioblastoma, which were infiltrated by reactive astroglia. However, in glial tumours, neither necrosis nor infiltration by reactive astroglia had any clear implication for GFA content. This probably means that GFA is produced by the malignant cells within the tumour mass and not simply by reactive astrocytes.

This work was supported by The Danish Medical Research Council.

### REFERENCES

BIGNAMI, A. & DAHL, D. (1974) Astrocyte-specific protein and radial glia in the cerebral cortex of newborn rat. *Nature*, **252**, 55.

DAHL, D. (1976) Glial fibrillary acidic protein from bovine and rat brain. Degradation in tissues and homogenates. *Biochim. Biophys. Acta*, **420**, 142.

DAHL, D. & BIGNAMI, A. (1973) Immunochemical and immunofluorescence studies of the glial fibrillary acidic protein in vertebrates. *Brain Res.*, **61**, 279.

DECK, J. H. N., ENG, L. F., BIGBEE, J. & WOODCOCK, S. M. (1978) The role of glial fibrillary acidic protein in the diagnosis of central nervous system tumours. *Acta Neuropathol. (Berl.)*, **42**, 183.

DELPECH, B., DELPECH, A., VIDARD, M. N. & 4 others (1978) Glial fibrillary acidic protein in tumours of the nervous system. *Br. J. Cancer*, **37**, 33.

DITTMANN, L., AXELSEN, N. H., NØRGAARD-PEDERSEN, B. & BOCK, E. (1977) Antigens in human glioblastomas and meningiomas: Search for tumour and onco-foetal antigens. Estimation of S-100 and GFA protein. *Br. J. Cancer*, **35**, 135.

DUFFY, P. E., GRAF, L., HUANG, Y.-Y. & RAPPORT, M. M. (1979) Glial fibrillary acidic protein in ependymomas and other brain tumours. *J. Neurol. Sci.*, **40**, 133.

ENG, L. F., VANDERHAEGHEN, J. J., BIGNAMI, A. & GERSTL, B. (1971) An acidic protein isolated from fibrous astrocytes. *Brain Res.*, **28**, 351.

HARBOE, N. & INGILD, A. (1973) Immunization, isolation of immunoglobulins, estimation of antibody titre. *Scand. J. Immunol.*, **2**, Suppl. 1, 161.

JACQUE, C. M., VINNER, C., KUJAS, M., RAOUL, M., RACADOT, J. & BAUMANN, N. A. (1978) Determination of glial fibrillary acidic protein (GFAP) in human brain tumours. *J. Neurol. Sci.*, **35**, 147.

KERNOHAN, V. W., MABON, R. F., SWIEN, H. J. & ADISON, A. W. (1979) A simplified classification of gliomas. *Proc. Mayo Clin.*, **24**, 71.

MØLLER, M., INGILD, A. & BOCK, E. (1978) Immunohistochemical demonstration of S-100 protein and GFA protein in interstitial cells of rat pineal gland. *Brain Res.*, **140**, 1.

SCHACHNER, M., HEDLEY-WHYTE, E. T., HSU, D. W., SCHOONMAKER, G. & BIGNAMI, A. (1977) Ultrastructural localization of glial fibrillary acidic protein in mouse cerebellum by immunoperoxidase labelling. *J. Cell Biol.*, **75**, 67.

UYEDA, C. T., ENG, L. F. & BIGNAMI, A. (1972) Immunological study of the glial fibrillary acidic protein. *Brain Res.*, **37**, 81.

VAN DER MEULEN, J. D. M., HOUTHOFF, H. V. & EBELS, E. J. (1978) Glial fibrillary acidic protein in human gliomas. *Neuropath. Appl. Neurobiol.*, **4**, 177.

WEEKE, B. (1973) Rocket immunoelectrophoresis. *Scand. J. Immunol.*, **2**, Suppl. 1, 37.